# *Chlamydia trachomatis*: From Urogenital Infections to the Pathway of Infertility

**DOI:** 10.3390/genes16020205

**Published:** 2025-02-07

**Authors:** Rafaela Rodrigues, Carlos Sousa, Alberto Barros, Nuno Vale

**Affiliations:** 1 PerMed Research Group, RISE-Health, Faculty of Medicine, University of Porto, Alameda Professor Hernâni Monteiro, 4200-319 Porto, Portugal; rafaela24sofia@hotmail.com (R.R.); carlos.sousa@unilabs.com (C.S.); abarros@med.up.pt (A.B.); 2 Molecular Diagnostics Laboratory, Unilabs Portugal, Centro Empresarial Lionesa Porto, Rua Lionesa, Leça do Balio, 4465-671 Matosinhos, Portugal; 3 RISE-Health, Department of Pathology, Faculty of Medicine, University of Porto, Alameda Professor Hernâni Monteiro, 4200-319 Porto, Portugal; 4 Centre for Reproductive Genetics Alberto Barros, 4100-012 Porto, Portugal; 5 RISE-Health, Department of Community Medicine, Health Information and Decision (MEDCIDS), Faculty of Medicine, University of Porto, Rua Doutor Plácido da Costa, 4200-450 Porto, Portugal

**Keywords:** genomic landscape, polymorphisms, sexually transmitted infections, urogenital tract, *Chlamydia trachomatis* pathogenesis, inflammation, immune response, infertility

## Abstract

*Chlamydia trachomatis* (CT) is a major cause of sexually transmitted infections (STIs) worldwide, with significant implications for reproductive health. The bacterium’s genome contains highly polymorphic regions, influencing both the type and severity of infections. These genetic variations, particularly those occurring in the major outer membrane protein (MOMP) gene, are critical for classifying the bacterium into distinct serovars and enable CT to adapt to diverse host environments, contributing to its immune evasion, persistence, and pathogenicity. Persistent or untreated urogenital infections can lead to chronic inflammation, tissue damage, and pelvic inflammatory disease, ultimately increasing the risk of ectopic pregnancy, spontaneous abortion, and infertility. This review consolidates current knowledge on the genetic diversity of CT, its potential role in modulating infection outcomes, and its immune evasion mechanisms. By integrating scientific evidence linking chlamydial infections to infertility, we underscore the urgent need for targeted research to address this critical public health challenge.

## 1. Introduction

**Figure 1:**
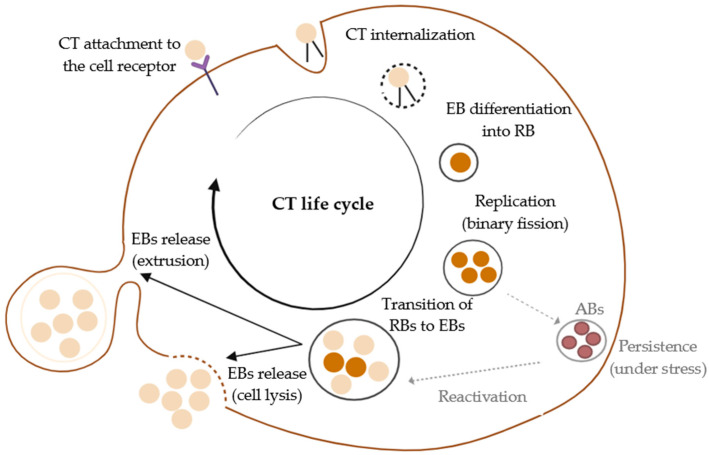
CT lifecycle. Herein is shown that the bacterium can transit into two distinct stages. The elementary body (EB), its infectious and non-replicative form, can survive outside of the host cell and is adapted to pursuit for a suitable host cell to initiate the infection. Later, with contact with a suitable host cell, EBs can reach the cell cytoplasm by adhesion and internalization into a vacuole. In this phase, the pathogen’s EB differentiates into the replicative form, the reticulate body (RB). Bacterium replication occurs using the host’s cell machinery and metabolites. Afterall, the RBs transit back into EBs that are able to infect other cells to continue the pathogen lifecycle. In detail, EBs can be released outside of the host cell through (1) lysis or (2) extrusion. Of note, under stress conditions, RBs can dedifferentiate into aberrant bodies (ABs), and the infection persists until the conditions become favorable for the lifecycle of the bacterium (reactivation of ABs to EBs). Figure created using BioRender https://www.biorender.com/ (accessed on 12 November 2024).

Sexually transmitted infections (STIs) are a major public health concern in developed countries due to their significant impact on communities, particularly because of the complications that can arise if they are not diagnosed and treated in a timely manner. Among these, chlamydial infections stand out as the most common bacterial STI. According to the European Centre for Disease Prevention and Control (ECDC), 216,508 confirmed cases of *Chlamydia trachomatis* infection were reported in 27 EU/EEA countries in 2022. Moreover, the ECDC reports a steady increase in these rates since at least 2018, highlighting the growing prevalence of this pathogen in recent years [1]. CT is an obligate intracellular pathogen of eukaryotic cells capable of infecting several different cell types. It is a simple parasite, composed of DNA, RNA, and ribosomes, and enclosed by a cell wall [2]. This Gram-negative pathogen is capable of causing a diversity of pathogenicity in humans, as it exhibits different tissue tropisms depending on certain genetic characteristics [3]. Specifically, the bacterium could cause extragenital or urogenital tract infections; however, in this manuscript, we will give emphasis to the urogenital infections, which will be explored in greater detail. The majority of CT urogenital infections are clinically asymptomatic, and the infection has no specific symptoms [4]. As a result, the infection transmission rate is high and, when diagnosed, it is often already associated with urogenital and reproductive tract complications, influencing patient morbidity [5]. In terms of cell biology, obviously, this obligate intracellular bacterium is totally dependent on host cell biosynthetic machinery for its development [2,6]. The developmental cycle is biphasic, constituted by two different morphological stages of CT: the elementary body (EB) and the reticulated body (RB) [7]. In detail, in terms of time, after infecting one cell, within 8 h, the bacterium is able to initiate replication within the host cell, and usually, in 48 to 72 h, the development cycle is completed, and the bacteria are ready to disseminate from this first host cell to other surrounding cells [8,9]. The first step of its biphasic development cycle begins with the EB, the infectious form, which is not able to replicate but is essential to promoting bacterium survival. It can bind to the host cell receptor through bacterial adhesins, and the invasion process starts [10]. After the contact, EBs are internalized into the inclusion bodies, a type of cellular vacuole, and gene transcription is initiated, ensuring all conditions for the bacteria lifecycle to occur and establishing an anti-apoptotic state in the host cell [7,8]. With the bacterial synthesis initiation, EBs differentiate into the reticulated body (RB), an actively replicating form, which then initiates replication at an alarming rate within the inclusion bodies (Figure 1) [9]. After completing these replication cycles, RBs re-differentiate back into EBs, which then can be released through host cell lysis or through extrusion to infect other cells, continuing the infection process [11]. Importantly, the RBs can enter a persistent non-replicative state under adverse conditions and cellular stress, dedifferentiated into aberrant bodies (ABs), leading to a chronic infection that is difficult to diagnose and treat [12]. In this state of persistence, the pathogen evades immune detection because it is less metabolically active and less susceptible to antibiotics [13].

In fact, without an effective vaccine development, antibiotics are the only treatment used to treat these infections [14]. There are many classes of antibiotics, among which macrolide and tetracycline are the most prescribed ones—specifically, the drugs azithromycin and doxycycline, respectively. These most effective anti-chlamydial drugs act by inhibiting bacterial protein synthesis through distinct mechanisms [13,15]. In detail, macrolide drug action occurs through its binding to a specific region of the 50S ribosomal subunit of the ribosome, which physically blocks the channel, inhibiting protein synthesis [15,16,17]. Tetracycline drugs can interfere in two different pathways: it not only can increase the CT membrane permeability, leading to bacterium death, but also it can bind to the 30S subunit of the ribosome, blocking tRNA binding and ultimately disrupting protein synthesis [15,16]. The proper use of antibiotics is a crucial factor in preventing bacteria from entering a protective state [18]. Importantly, in the persistent form, CT can be reactivated when intracellular conditions change, or in other words, when the factors that have triggered persistence are eliminated. For example, CT reactivation can occur upon the removal of stress conditions, such as nutrient deprivation, oxidative stress, or the cessation of antibiotic treatment [8,12,18]. Also, this chronic condition in the female genital tract causes long-term exposure of the reproductive organs to the bacterium, leading to inflammation and scarring [19]. This is the leading cause of tubal factor infertility, a condition in which the fallopian tubes are congested, avoiding spermatozoa from reaching an egg for fertilization or preventing an embryo from reaching the uterus for pregnancy [20]. Also, chronic CT infection can be associated with pelvic inflammatory disease (PID), preterm birth, endometritis, and perihepatitis [21,22]. Indeed, PID, which is a female heterogenous syndrome possibly associated with infertility, ectopic pregnancy, and chronic pelvic pain, is also a well-established risk factor for infertility [23,24,25]. The fact that CT is one of the leading causes of PID and that pelvic inflammatory disease is a syndrome known to contribute to infertility, are other scientifically based validations to hypothesize this infection as a potentially significant risk factor for infertility [24].

It is also important to emphasize that the diagnose of CT infection in men is very relevant, not only because they provide an important reservoir of bacteria for new and recurrent infections in women but also because they may experience serious adverse outcomes as well [26]. In detail, the most common outcomes of prolonged CT urogenital infection in men are urethritis, epididymis, prostatitis, and Reither’s syndrome [27,28]. Infertility could also be a consequence in this gender, as we will further explore later [29,30].

In this review, we aim to present a comprehensive analysis of the current understanding of the relationship between chlamydial infection and the pathways to infertility in males and females. Additionally, we seek to elucidate whether the bacterium’s genetic characteristics contribute to differing variation in clinical outcomes, including the heterogeneity of the infection’s worst consequences. At some point, we try to understand whether each different infection itself with a particular strain of CT may provide some clues regarding the subsequent infection outcome, seeking the possibility of a personalized approach depending on each infection in a specific individual.

## 2. *C. trachomatis* Genome

**Figure 2:**
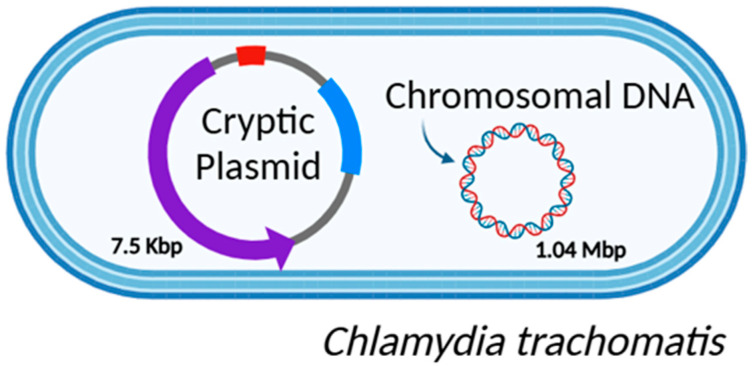
Schematic representation of CT genome. In particular, the virulent plasmid of approximately 7.5 Kbp and the circular chromosome of 1.04 Mbp. Figure created using BioRender https://www.biorender.com/ (accessed on 4 November 2024).

As detailed in our previous work, early research on CT biology was rudimentary, relying primarily on basic cell culture, serology, and immunohistochemistry techniques [31]. However, recent technological advances, including high-resolution microscopy and the development of omics approaches, have significantly advanced the field of research into the genetic diversity of bacteria [32]. In particular, the advent of new sequencing methodologies using molecular techniques has been crucial for studying pathogens like CT, which is challenging to manipulate in vitro, mostly due to its unique biology and developmental lifecycle [33]. These molecular advancements have enabled researchers to thoroughly characterize this pathogen, as well as many others. Researchers demonstrated that CT has a small genome (Figure 2) due to the long-term adaptation to an obligate intracellular lifestyle constituted by a circular chromosome of 1.04 Mbp containing 900 to 1500 coding sequences. Additionally, it is constituted by a highly conserved chlamydial virulent plasmid of approximately 7.5 Kbp, which may be present in several copies [34,35,36].

Of note, most of the bacterial variability is attributed to single nucleotide polymorphisms (SNPs), influencing virulence and tissue tropism and leading to CT infections in particular regions of the human body. Therefore, CT is classified into different biovars: trachoma, epithelial ano-urogenital, and LGV biovars [37]. This distinction is extremely important for the correct treatment and management of CT infection. In detail, the trachoma biovar infects conjunctival epithelial cells, causing trachoma, the leading cause of preventable blindness. The ano-urogenital biovar, the most frequent infection of both sexes, is responsible for severe complications related to the reproductive tract, and it is able to infect many other epithelial cells, such as those in the respiratory, gastrointestinal, and ocular (non-trachoma) tracts. Also, it can cause neonatal infections through vertical transmission. Finally, the LGV biovar is much more invasive than the others, and it can cause lymphogranuloma venereum (LGV), a systemic infection that starts in the lymphatic tissue and can cause irreversible sequelae if not treated adequately [38,39,40]. These biovars are further divided into different serovars based on the variability of the *ompA* gene, which confers specific characteristics to the bacterium [40]. The *ompA* gene encodes the major outer membrane protein (MOMP) of the bacterium and exhibits polymorphism through single nucleotide polymorphisms (SNPs) [32,41]. Based on these polymorphisms, the trachoma biovar includes serovars A to C. Serovars D to K are the most frequent and, as mentioned before, can infect all the following tracts: urogenital, pharyngeal, conjunctiva, and anorectal [42,43]. Lastly, the LGV biovar comprises serovars L1 to L3 (Table 1) [9].

It is interesting to understand that from the molecular point of view, there is genomic conservation between all the different CT serovars in more than 98%. Notwithstanding, despite these genotypes’ similarity, phenotypically there are evident differences, specifically in terms of infection outcomes and bacteria tissue tropism [32]. Therefore, is it clear that minor genetic variations provide key characteristics of the bacterium, influencing infection pathobiology. In detail, to date, these key variations are described as occurring due to SNPs, insertion or deletions (INDELs) in genomic DNA, or bacterium plasmid variations. Also, epigenetic factors could contribute to the differences in infection outcome, specially through a mechanism of miRNA regulation [44].

### 2.1. CT Genomic DNA

The small, circularized genome of CT is composed of some important elements—specifically, the plasticity zone (PZ), T3SS, Incs, Pmps, ompA, tarP, and Trp operons [32,45]. Some of these are represented in Table 2 and have great significance in the bacterium virulence and tissue tropism, especially the PZ genomic region, which is highly variable, containing more than 45 genes, partial Trp operons, and the cytotoxin gene (tox) [46]. This particular tox gene is informative, identifying CT tissue tropisms. Tox open reading frames (ORFs) can be intact in urogenital strains, truncated in ocular strains, or absent in LGV strains [47]. Additionally, the T3SS region is one of the most crucial elements for the translocation of effector proteins into the host cell. It contains 20 to 25 genes encoding for highly conserved apparatus scaffolding, inner membrane components, extracellular needles, chaperons, and various effectors [9,48]. TarP is one of these T3SS effectors. It is responsible for actin cytoskeleton remodeling, contributing to the entry of CT into the host cell. Variations in this gene sequence, translated into distinct N-terminal tyrosine-repeat units and C-terminal binding domains, are theorized to contribute to bacterium virulence and tissue tropisms [49]. Another relevant region is the one encoding for Inc proteins, which may be associated with tissue tropisms and disease severity between LGV and trachoma biovars of CT [9]. In detail, when some variations in amino acids occur in regions of Inc proteins, which are exposed to the host cytoplasm during the infection, they may influence host–pathogen interactions and immune recognition [49,50]. Almeida and colleagues found that LGV strains present a characteristic gene expression pattern in CT058, CT192, and CT214 of the Inc region, mediated by specific gene mutations in CT192 and CT214, and by post-transcriptional regulation of CT058 transcripts, which may be associated with tissue tropism targeted more towards macrophages [50]. In addition, pmp genes encode for a group of potential virulence factors: polymorphic membrane proteins (Pmps), a family of surface-exposed membrane proteins. To the best of our knowledge, the exact function of Pmps remains unclear. Notwithstanding, it is hypothesized that they are implicated in attachment, invasion, and immune evasion processes [51]. Interestingly, although genome reduction is obvious for almost every class of proteins of the distinct species of Chlamydia (genus), there is expansion of the Pmp family, which holds distinctive significance [52,53]. The OmpA gene also plays a key role in the bacterium genome, as it is highly polymorphic and encodes for the chlamydial major outer membrane protein (MOMP), a surface-exposed porin protein. This gene is composed of four highly polymorphic variable sequences (VSs), which are flanked by five constant sequences (CSs) [54]. As described before, it is used for CT serovar classification [55].

Indeed, there are several genomic regions where occur variation could occur, resulting in distinct infection and outcomes.

### 2.2. CT Plasmid Diversity

Chlamydial plasmid contains eight coding sequences (CDSs), known as plasmid glycoproteins (pGPs 1 to 8), whose role is to facilitate the infection ascension, induce inflammation, and contribute to the extrusion process, as well as four tandem repeat sequences [56,57,58]. Each CDS encodes distinct proteins, including integrase (for plasmid replication), helicase, a secreted virulence protein (associated with persistent infection), a transcriptional regulator for genes located both on the plasmid and on the chromosome, glycogen synthase, infectivity factors, and partitioning proteins [56,59,60,61].

In the diagnosis of *C. trachomatis* infection, the CDSs of the bacterium’s plasmid were valuable targets for testing due to their stability and high copy number [62]. Nonetheless, the emergence of a mutant strain from Sweden, which contains a 377 bp deletion in CDS1 of the plasmid, has led to false negative test results, making this target unsuitable for reliable detection [63]. Of note, this variant was characterized using whole genome sequencing (WGS) and has since been occasionally reported in other countries [64]. Additionally, other variants, resulting from mutations in CT plasmid, such as the Finnish New Variant, have been reported recently, emphasizing the need for more sensible diagnosis using not only plasmid-based targets for bacterium detection [62].

Despite CT plasmid not being essential for bacterium survival, it is crucial to attribute distinct virulence. In vivo studies with a CT strain carrying plasmid deletion have showed its significance to pathology and inflammation [65]. Sigar et al. demonstrated that without the plasmid, there was a virulence attenuation and a lower inflammatory response, concluding that the type of plasmid influences the disease outcome [66]. Additionally, it is known that in stress conditions, the copy number of plasmids increases; however, not all the functions of this bacterial entity are yet understood [58].

### 2.3. Patho-Epigenetics in Chlamydial Infection

**Figure 3:**
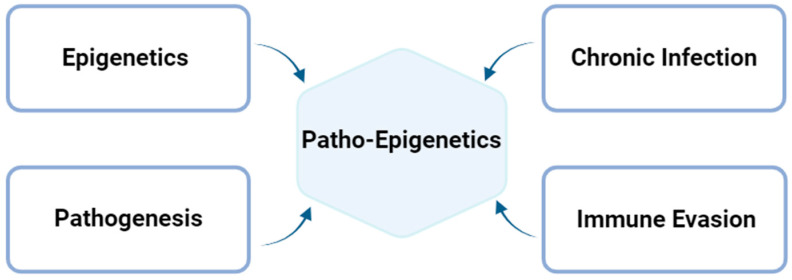
Patho-epigenetics associated with *C. trachomatis* infection. CT invades the host and can establish long-term, persistent infections. This chronic infection has the potential to enhance epigenetic changes and increase the bacterium’s ability to evade the immune system. Together, these dysregulations contribute to the epigenetic alterations associated with the pathogenesis driven by CT infection. Figure created using BioRender https://www.biorender.com/ (accessed on 15 November 2024).

Epigenetic modifications are alterations affecting gene expression that occur in the genome without changing the DNA sequence [66]. The most common types of these modifications include DNA methylation, post-translational modifications of the histones, and gene silencing via non-coding RNA, such as miRNAs or siRNAs [67]. These modifications can be influenced by environmental factors, lifestyle, or infections caused by specific pathogens, such as viruses and intracellular bacteria. Specifically, patho-epigenetics is a term that encompasses the development of a disease (pathogenesis) prompted by epigenetic dysregulation [68]. In the specific case of prolonged chlamydial infection, this designation arises from distinct factors that contribute to the patho-epigenetic establishment, as represented below (Figure 3). Where CT invades the host, it is well known that there is a possibility that it could be a long-term infection [7]. This chronic infection could potentially increase the epigenetic changes, as well as the bacterium’s ability to evade the immune system [69]. Altogether, these dysregulations contribute to epigenetic changes associated with the pathogenesis created by the bacterium itself [68].

In fact, there is evidence that intracellular bacteria can trigger epigenetic changes in the host cells to guarantee their maintenance, replication, and transmission [70,71]. CT has been reported to trigger specific epigenetic modifications in the host cell that are linked to its pathogenic effects [72]. One key role of the bacterium is the regulation of miRNAs, which are small non-coding RNA molecules that play a critical function in controlling gene expression by binding to messenger RNA (mRNA) and preventing the production of certain proteins [73]. miRNAs are essential for processes such as cell proliferation, immune response, and tissue development [74]. CT infection disrupts normal miRNA processing, leading to dysregulation of several important pathways [75]. This disruption occurs through RNA interference (RNAi), a cellular mechanism where miRNAs and other small RNAs silence specific genes after transcription [75,76]. RNAi can result in either mRNA degradation or the inhibition of protein translation, depending on the pathway involved [77]. This disruption of gene regulation is significant and requires careful exploration. In detail, two distinct mechanisms are thought to be involved in this process [73]. The first is the inactivation of the Dicer, an essential component in generating miRNAs and whose role is to cleave precursor RNA molecules to mature miRNAs, which are crucial for several cellular processes, such as cell proliferation and immune response [78,79,80]. Indeed, there are some reports showing that Dicer modifications are involved in infertility in in vivo mouse models [81]. Additionally, the second mechanism involves Argonaute 2 (Ago2), a key protein in RNAi [73]. During CT infection, Ago2 levels are reduced, which significantly impacts fertility by affecting key processes in the female reproductive tract. This dysregulation may lead to infertility and other pathological conditions, such as tumorigenesis [80]. Thus, it could be interesting to focus some future studies on the epigenetic landscape to further understand the potential of these modifications in human infections in order to uncover potential biomarkers associated with chlamydial infection severity and infertility.

## 3. Long-Term Chlamydial Urogenital Infection and Infertility

Despite the significant rate of spontaneously resolved chlamydial infections, specifically with 50% of cases resolving within one year, 82% resolving within two years, and 95% of cases resolving within three years, there is still a risk of developing severe complications [82,83]. One of the potentially most significant long-term consequences of chlamydial infection is infertility [84]. The repercussions of infertility extend beyond the individual, affecting psychological well-being, the couple’s stability, societal dynamics, and even the economy of countries [85].

According to the World Health Organization (WHO), infertility affects approximately one in six individuals globally [86]. This fact underscores the urgent need for research to understand the factors contributing to this trend of increasing rate, highlighted by Centers for Disease Control and Prevention (CDC) [87]. The prevention of infertility is extremely important; thus, given the far-reaching implications of chlamydial infection on reproductive health, it is crucial to examine its role in infertility to better understand the extent of its influence [88,89]. In the literature, there is accordance that if this bacterium goes undiagnosed or if there are repeated infections, it can lead to severe consequences in the female upper genital tract [90,91,92]. Also, in males, evidence suggests that the infection may influence their reproductive capability [93]. Notwithstanding, it is important to reinforce that the association between chlamydial infection and infertility is still under scientific debate, as a direct causal relationship is not well documented in the literature, and the majority of the studies have significant biases. Furthermore, the ECDC has published a document defending that a significant proportion of chlamydial infections in women do not lead to PID, and that even when PID does occur, it does not always result in infertility [94].

Chlamydial genital infections are often asymptomatic, meaning that the infected individuals are not diagnosed and treated properly, thus transmitting the infection to their sexual partners and becoming more susceptible to long-term complications from the infection [95]. Importantly, this infection affects mostly younger adults (<25 years old), having a potentially high impact on their fertility capability if not detected and treated in a timely manner [96]. This persistent presence of the pathogen in the genital organs leads to a chronic immune response, characterized by the increased production of pro-inflammatory cytokines, creating a pro-inflammatory milieu favorable to epithelial cell damage [91,97,98]. Subsequently, this pro-inflammatory response gradient could trigger serious repercussions for women’s reproductive health, including PID, chronic pelvic pain, adverse pregnancy outcomes, infertility, and even tumorigenesis [5,99].

**Figure 4:**
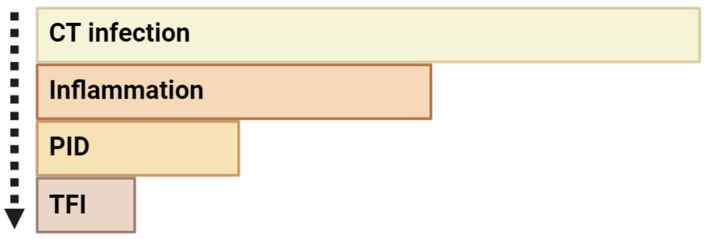
Possible stages that could be associated with chlamydial infection leading to female infertility development. Initially, CT infection occurs. Then, it could trigger an inflammation process, which subsequently could lead to pelvic inflammatory disease (PID). This latter condition, in some cases, could potentially trigger tubal factor infertility. Of note, the arrow indicates the sequence of events. The rectangles size for each condition represents the relative frequency of each condition compared to the others. Note that the rectangle sizes are for illustrative purposes only and are not drawn to scale. Figure created using BioRender https://www.biorender.com/ (accessed on 1 November 2024).

Indeed, infertility may result from scar formation and occlusion of the fallopian tubes, a possible consequence of chlamydial infection (Figure 4) [20,100]. In detail, during this infection, the bacterium can ascend from the lower genital tract, reaching the uterus, fallopian tubes, and ovaries [101]. This process triggers an exacerbated immune response, responsible for creating a pro-inflammatory cascade that causes significant tissue damage and fibrosis, ultimately leading to pelvic inflammatory disease (PID) [102,103,104]. Then, scarring and blocking of the fallopian tubes leads to a condition referred to as tubal factor infertility (TFI) [105]. In most cases, TFI arises from inflammation of the epithelial tissues of the fallopian tubes and pelvic–peritoneal adhesions, which results in the inability for fertilization or embryo implantation [106]. This means that one of the possible underlying mechanisms of CT infection causing infertility is the intensified host immune response, which establishes an inflammatory milieu favorable to tissue damage in the reproductive organs [92].

In fact, first, it is important to understand how the immune system of the host reacts when the chlamydial infection occurs, as it must be our “defense shield” against external agents such as CT [107,108]. Briefly, through cell surface and endosomal receptors, the immune system is aware that an infection occurred. In the cervicovaginal mucosa, microbiota—specifically, lactobacillus—confer an important defense to this region [109]. Therefore, imbalance in women’s vaginal microbiota confers a predisposition to vaginal infections [110]. If conditions are reunited, CT surpasses this barrier and the infection process begins. Host immune responses against the bacterium primarily occur through the innate immune system, the first line of defense, providing a rapid, non-specific response to pathogens. No prior exposure is required to recognize the pathogens, and mast cells are among the first barrier that CT has to surpass [111,112]. In addition, dendritic cells play a crucial role, not only by capturing and processing antigens but also by triggering the adaptative immune response, presenting the antigen to T cells through the molecules of major histocompatibility complex class I or class II (MHC-I and MHC-II, respectively) [113]. Subsequently, activated T cells (effector and memory cells) and NK cells produce IFN-γ to activate macrophages, whose role is to clear the infection [114,115,116,117]. Of note, antigen-specific immunity, producing immunity via B-cell involvement, also forms part of the adaptative immune response, which is also important for fighting against this pathogen on a secondary level [111,117].

**Figure 5:**
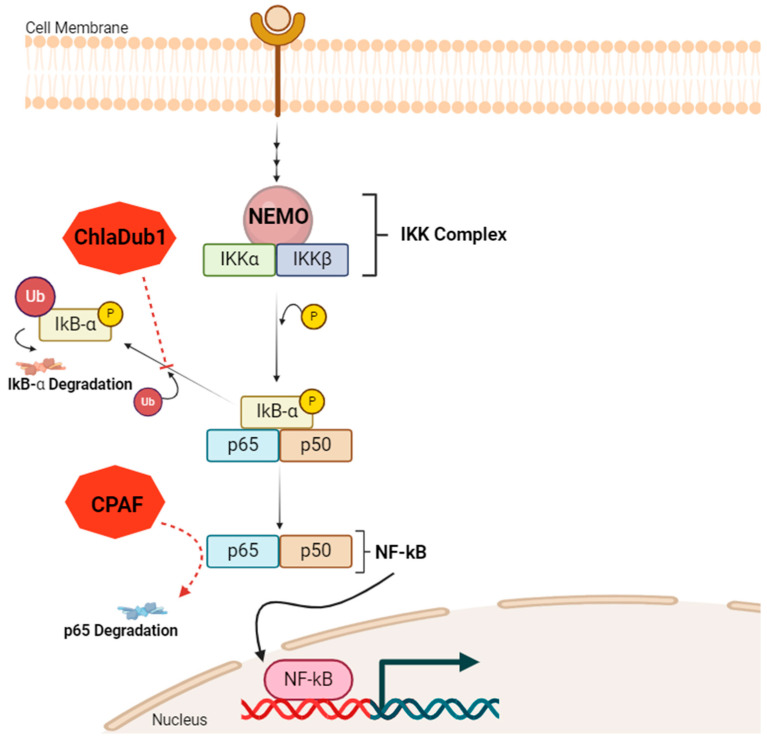
NF-kB pathway dysregulation in chlamydial infection. ChlaDub1, a deubiquitinating protease from CT, acts downstream of the IKK complex, which consists of NEMO, IKKα, and IKKβ. The IKK complex phosphorylates the NF-kB inhibitor, IkB-α, targeting it for ubiquitination and subsequent degradation. ChlaDub1 inhibits this process by preventing IkB-α ubiquitination and posterior degradation, thereby blocking the release of the NF-kB transcriptional complex (composed of p65 and p50). Additionally, CT could also interfere at another stage, through CPAF, promoting p65 degradation and destroying the NF-kB complex, which cannot translocate to the nucleus to transcribe target genes associated with the inflammatory response. Figure created using BioRender https://www.biorender.com/ (accessed on 13 November 2024).

The mechanisms of CT to evade the host immune system are diverse. There is recent in vitro evidence that NF-kB, a transcription factor that plays an important role in the regulation of inflammatory response, immune cell activation, and the production of some cytokines, is actively inhibited during CT infection [118]. Of note, NF-kB transcription factor is a complex formed by two distinct units: p65 and p50 [119]. Le Negrate and colleagues described two possible mechanisms underlying NF-kB pathway dysregulation (Figure 5) [120]. In their study, the authors demonstrated that a deubiquitinating protease from the bacterium ChlaDub1, which is expressed by CT in infected cells, has the ability of NF-kB pathway inhibition, acting downstream of the IKK complex, which is composed of three different units: NEMO, IKKα, and IKKβ [119]. In detail, ChlaDub1 blocks IkB-α degradation through the impairment of its ubiquitination [120]. Therefore, the transcription factor NF-kB is not able to migrate to the cell nucleus. Another mechanism is p65 degradation through a chlamydial protease or proteasome-like activity factor (CPAF), destroying a subunit of NF-kB [121].

Moreover, CT can also evade the host immune system using other mechanisms, such as inhibiting the cytokines and chemotactic molecules produced by epithelial cells, interfering with antigen-presenting cell (APC) functions, or downregulating MHC class I and II molecules [122]. Therefore, by reducing the antigen-presenting capacity of the APCs and subsequently reducing immune cell recruitment to the region of the infection, CT leads to lower immune system activity and capacity to clear the infection [122,123].

CT intracellular lifecycle allows the bacterium to remain protected within the inclusion body, shielding it from humoral immunity [111]. Additionally, recent findings showed that CT has another way to escape the immune system: by RBs, which can invade other host cells through nanotubes (TNTs), avoiding the extracellular microenvironment, which is in reach of immune cells and molecules, and thus increasing the bacterium’s survival chances [124]. In addition, the bacterium could enter a state of persistence and latency, helping it survive under less favorable conditions in the microenvironment [12]. CT also evades host cell apoptosis, which is crucial for its replication and survival [111]. Moreover, the bacterium can modulate the immune response, leading to an exacerbated inflammatory response that may cause organ damage. During this process, symptoms are often absent, which can result in irreversible reproductive damage, such as infertility [125,126].

In fact, currently, the mechanisms of host immune evasion by CT still need to be further explored to understand the interaction between the immune cells and the bacterium to better combat the infection [111]. It is also important to highlight that pathogens other than CT can cause reproductive organs damage and that these infections can co-occur in the same host, such as *Neisseria gonorrhoeae* infection, HPV, HIV, syphilis, *Trichomonas vaginalis*, bacterial vaginosis, and *Mycoplasma genitalium* [5,125,127,128,129]. Therefore, these co-infections could potentially increase the risk of severe sequelae, such as tumorigenesis and infertility, highlighting the relevant role of multi-test diagnosis of STIs [5].

In men, the association between chlamydial infection and infertility it is not as well studied as it is in women. Notwithstanding, CT infection in males can evidently lead to inflammatory conditions such as urethritis, epididymitis, or, less commonly, prostatitis [130,131,132,133]. Furthermore, evidence suggests that CT infection may affect spermatogenesis—in detail, the bacterium can penetrate inside the spermatozoa, affecting the quantity and quality of sperm [82,83,134]. Additionally, some authors argue that the bacterium interacts with sperm, inducing oxidative stress that leads to apoptosis of the cells [135]. Indeed, CT could also cause obstructive azoospermia, interfering with spermatozoid motility [135,136].

Recent studies have provided additional insights into these mechanisms. Stojanov and colleagues have proposed the “intracellular bacterial hypothesis”, which suggests that intracellular bacteria like *C. trachomatis* can impair male fertility by interacting directly with sperm cells, inducing oxidative stress and triggering apoptosis. This aligns with findings that CT infection can alter sperm motility and viability, potentially affecting overall fertility [137]. In line with this, Wang et al. (2021) highlighted the role of oxidative stress and autophagy in the pathogenesis of bacterial infections, including CT, as contributors to male infertility. These findings suggest that prolonged CT infections may interfere with sperm function beyond the standard sperm parameters assessed in routine analysis, potentially affecting processes like fertilization or implantation [138].

Moreover, Moazenchi 2018 conducted a comprehensive study demonstrating the significant impacts of CT infection on sperm parameters, including motility and morphology. Importantly, through flow cytometry, the authors also assessed the DNA fragmentation index of the sperm. Finally, the authors suggest that DNA damage in sperm and seminal parameter alterations (concentration, morphology, viability, and motility) might be directly associated with CT infection and infertility [139,140]. Interestingly, last year, Haratian and colleagues identified an SNP in the *DEFB126* gene (rs11467417), which is primarily expressed in the principal epididymal epithelial cells. This genetic variant may increase susceptibility to CT infection and subsequent infertility in men [141].

Therefore, based on cumulative evidence, it is possible to affirm that there may be an association between chlamydial long-term infections and male fertility impairment, although contradictory findings in the literature highlight the need for further investigation [142].

It is important to highlight that infertility is indeed a huge global health issue that carries profound economic and societal consequences [143]. According to Inhorn and Patrizio, a country’s economic burden associated with infertility includes direct healthcare costs, such as fertility treatments, and indirect costs, such as lost productivity and the psychological health impairment of individuals and their families [144]. As a consequence, infertility may also hinder the ability to maintain employment, potentially resulting in prolonged economic or financial challenges [145]. Given the significant personal and societal impacts, it is critical that countries prioritize investment in preventive measures for infertility cases, beginning with preventable factors such as STIs—particularly chlamydial infections. Preventive measures such as increased screening, early diagnosis, and treatment of chlamydial infections are cost-effective solutions that could alleviate both the financial burden on healthcare systems and the emotional suffering of those affected [146].

## 4. Conclusions and Future Directions

CT, the most common cause of bacterial STIs globally, has become a growing concern due to the sequelae that prolonged urogenital infection can leave [147]. One of the primary consequences of chlamydial urogenital infection is the activation of the host’s immune response, leading to the establishment of a pro-inflammatory milieu during prolonged infection. As a result, several complications may arise in both sexes, including chronic pain, urethritis, pelvic inflammatory disease (PID), infertility, pregnancy complications, epididymitis, prostatitis, and an increased risk of contracting other STIs. Moreover, there is evidence suggesting that these prolonged infections may be associated with events of oncogenesis. Of note, following the ECDC’s most recent data, CT infection rates are increasing. Therefore, these trends highlight the importance of primary and secondary prevention of STIs, with education on safe sex practices, and due to their asymptomatic presentation, the establishment of routine screening tests in all countries to detect infections in a timely manner and reduce their spread.

Throughout this article, we have reviewed the key biological and genetic aspects of CT, focusing on its genetic variations and their impact on infection outcomes. Indeed, genetic polymorphisms of CT are key determinants in the variability of bacterium virulence and tissue tropisms. Its unique lifecycle, characterized by the alternation between elementary bodies and reticulate bodies, allows CT to evade the immune system, contributing to persistent infections and making the clinical outcome of infection worse. These long-term urogenital infections influence the immune microenvironment and exacerbate inflammatory responses, contributing to tissue scarring and damage to the reproductive organs. Additionally, there is evidence in the literature suggesting that chlamydial infection could be associated with the development of infertility in both men and women. Notwithstanding, while the association between infection and reproductive sequelae is already demonstrated, the mechanisms underlying the infection and fertility impairment in both sexes remain unclear.

The analysis of the literature reveals that the association between infertility and CT infections has not been thoroughly investigated. Complications from infection may vary depending on the bacterial serovar, as well as individual factors of the infected person, such as the immune status and the vaginal flora. Notwithstanding, several important aspects remain unclear, including the self-clearance of CT infections and the interactions between the bacterium and immune system cells. There is lack of fundamental studies, and some of the findings came from research where numerous other variables, including co-infections, may act as confounding factors, influencing the conclusions drawn. It is also important to highlight that some studies exploring this association have some limitations that prevent an accurate generalization of their results. Additionally, biases may arise from differences in CT detection methods and infertility criteria across studies. Regarding the mechanisms underlying the infection, some findings are based on in vivo or in vitro models, which do not fully replicate the complexity of human infection. These limitations highlight the need for further studies with larger sample sizes and standardized criteria for bacterial detection and infertility assessment. Moreover, laboratory findings should be integrated with clinical studies and longitudinal data analysis to better understand the long-term outcomes of infection.

To address the gaps in knowledge, future research should also focus on the development of clinical applications aimed at improving the prevention, diagnosis, and treatment of this bacterial infection. In order to better manage the infection worldwide, vaccines against CT represent a promising avenue, particularly when administered to adolescents before their first sexual intercourse, as this could reduce the burden of asymptomatic infections and prevent complications such as infertility. Notwithstanding, despite the enormous efforts of the scientific community to develop a vaccine against this pathogen, no viable solutions of this type are currently available. In the absence of a vaccination plan, efforts can shift toward a more personalized approach to managing CT infections. While vaccines may one day be adapted to individual immune profiles or genetic markers, current advances in personalized medicine can already offer valuable alternatives. For instance, identifying genetic polymorphisms in CT and the host immune system can pave the way for targeted treatments based on host genetic markers or immune profiles. This approach enables a better understanding of individual susceptibility to CT infection and allows for therapies that improve treatment efficacy and reduce adverse effects.

These advances, coupled with a deeper understanding of CT pathogenesis, will be crucial in addressing the global burden of CT infection and improving reproductive health outcomes.

## Figures and Tables

**Table 1:** *Chlamydia trachomatis* biovars and serovars.

Biovar	Serovar
Trachoma	A
	B
	Ba
	C
Ano-urogenital	D
	Da
	E
	F
	G
	Ga
	H
	I
	Ia
	J
	Ja
	K
LGV	L1
	L2
	L2a
	L2b
	L3

**Table 2:** Role and characteristics of the most important genomic DNA elements of *C. trachomatis*.

Genomic Elements	Characteristics/Functions
Plasticity zone (PZ)	Contains more than 45 genes (*Trp* operons; *tox*). Highly variable among the distinct CT strains.
T3SS	Contains 20–25 genes encoding membrane-associated components and surface structures. Crucial role involved in the translocation of effector proteins into host cells.
TarP	T3SS effector. Involved in actin cytoskeleton remodeling, facilitating CT entry into host cells. Gene variations contribute to bacterial virulence and tissue tropism.
Incs	Associated with tissue tropisms and disease severity between LGV and trachoma biovars. Gene variations may influence host–pathogen interactions, tissue tropism, and immune recognition.
Pmps	Encodes for potential virulent factors and some membrane proteins. Potentially involved in CT attachment, invasion, and immune evasion processes.
ompA	Highly polymorphic. Encodes for the CT major outer membrane protein (MOMP), used for serovar classification.
